# Concomitant Assessment of Monocyte HLA-DR Expression and Ex Vivo TNF-α Release as Markers of Adverse Outcome after Various Injuries—Insights from the REALISM Study

**DOI:** 10.3390/jcm11010096

**Published:** 2021-12-24

**Authors:** Frank Bidar, Maxime Bodinier, Fabienne Venet, Anne-Claire Lukaszewicz, Karen Brengel-Pesce, Filippo Conti, Laurence Quemeneur, Philippe Leissner, Lionel K. Tan, Julien Textoris, Thomas Rimmelé, Guillaume Monneret, on behalf of the Realism Study Group

**Affiliations:** 1 EA 7426 “Pathophysiology of Injury-Induced Immunosuppression” (Université Claude Bernard Lyon 1—Hospices Civils de Lyon—bioMérieux), Joint Research Unit HCL-bioMérieux, Univeristé Lyon 1, 69003 Lyon, France; frank.bidar@hotmail.fr (F.B.); maxime.bodinier@biomerieux.com (M.B.); fabienne.venet@chu-lyon.fr (F.V.); anne-claire.lukaszewicz@chu-lyon.fr (A.-C.L.); Karen.BRENGEL-PESCE@biomerieux.com (K.B.-P.); filippo.conti@chu-lyon.fr (F.C.); julien.textoris@biomerieux.com (J.T.); thomas.rimmele@chu-lyon.fr (T.R.); 2 Anesthesiology and Critical Care Medicine, Edouard Herriot Hospital, Hospices Civils de Lyon, 69003 Lyon, France; 3 Immunology Laboratory, Hopital E. Herriot, Hospices Civils de Lyon, 69437 Lyon, France; 4 NLRP3 Inflammation and Immune Response to Sepsis Team, Centre International de Recherche en Infectiologie (CIRI), Inserm U1111, CNRS, UMR5308, Ecole Normale Supérieure de Lyon, Université Claude Bernard-Lyon 1, 69003 Lyon, France; 5 Sanofi Pasteur, Sanofi 1541 Avenue Marcel Mérieux, 69280 Marcy l’Etoile, France; Laurence.Quemeneur@sanofi.com; 6 BIOASTER, 40 Avenue Tony Garnier, 69007 Lyon, France; Philippe.LEISSNER@bioaster.org; 7 GSK, 980 Great West Road, Brentford, Middlesex TW8 9GS, UK; lionel.x.tan@viivhealthcare.com

**Keywords:** immunosuppression, monocyte, HLADR, TNF, LPS, sepsis, trauma, surgery

## Abstract

Intensive care unit (ICU) patients develop an altered host immune response after severe injuries. This response may evolve towards a state of persistent immunosuppression that is associated with adverse clinical outcomes. The expression of human leukocyte antigen DR on circulating monocytes (mHLA-DR) and ex vivo release of tumor necrosis factor α (TNF-α) by lipopolysaccharide-stimulated whole blood are two related biomarkers offered to characterize this phenomenon. The purpose of this study was to concomitantly evaluate the association between mHLA-DR and TNF-α release and adverse clinical outcome (i.e., death or secondary infection) after severe trauma, sepsis or surgery in a cohort of 353 ICU patients. mHLA-DR and TNF-α release was similarly and significantly reduced in patients whatever the type of injury. Persistent decreases in both markers at days 5–7 (post-admission) were significantly associated with adverse outcomes. Overall, mHLA-DR (measured by flow cytometry) appears to be a more robust and standardized parameter. Each marker can be used individually as a surrogate of immunosuppression, depending on center facilities. Combining these two parameters could be of interest to identify the most immunosuppressed patients presenting with a high risk of worsening. This last aspect deserves further exploration.

## 1. Introduction

The third sepsis conference in 2016 established a new definition for sepsis, which is now depicted as a life-threatening organ dysfunction caused by a dysregulated host response to infection [1]. This reflects our evolving understanding of sepsis pathophysiology since the host immune response in sepsis is complex, rapidly evolving over time and involves an initial excessive inflammation associated with a compensatory anti-inflammatory response [2,3]. When unbalanced, this response can lead to a delayed state of hypo-responsiveness known as sepsis-induced immunosuppression [3,4].

Given improvements in early sepsis detection and acute intensive care management, most patients now survive their initial septic insult, but a significant number do not fully recover immune functions and experience recurrent infections, which may contribute to increased morbidity or mortality [5,6]. Marked immunosuppression has been partially described in patients admitted to the intensive care unit (ICU) for severe trauma and major surgery [7,8]. A better understanding of the putative role of immune alterations in clinical worsening after an injury may enable the use of agents that stimulate the immune system as a potential therapeutic strategy to reduce morbidity and mortality of critically ill patients [9,10]. Because the immune response during the ICU course can vary among patients and over time, it is necessary to correctly identify the patients who will most benefit from these treatments [11]. The development of reliable biomarkers to identify individuals with clinically meaningful immunosuppression is thus required [9,12].

Among the variety of candidate biomarkers available to assess immunosuppression, two are interrelated since both focus on monocytes: expression of human leukocyte antigen DR on circulating monocytes (mHLA-DR) and ex vivo tumor necrosis factor α (TNF-α) release of lipopolysaccharide (LPS)-stimulated whole blood [13]. The diminished mHLA-DR expression was proposed as a reflection of monocyte deactivation in critically ill patients. It is considered a reliable marker of immunosuppression as it has been extensively studied in many clinical settings and repeatedly associated with an increased risk of nosocomial infection and mortality [3,14]. The measurement of mHLA-DR expression is a rapid, non-expensive and reproducible technique, but it requires the use of flow cytometry performed within 4 h of blood sampling, which may not be available routinely in all centres. TNF-α release upon ex vivo LPS challenge is a functional test to assess monocyte pro-inflammatory cytokine production capacity [15]. After a severe injury, a reprogramming of monocytes is observed, which results in a decreased TNF-α release capacity. This hypo-responsiveness to a second challenge led to the concept of endotoxin tolerance [16] and supported the use of TNF-α release as a reliable read-out of monocyte functionality. Decreased TNF-α release was also reported to predict patients’ outcomes in ICU [17]. However, reported results are more heterogeneous than those of mHLA-DR [18,19,20]. This may be owed to the fact that TNF-α release measurement is less standardized [11,19] and is based on whole blood that contains various types of leukocytes.

Only a few studies simultaneously assessed the performances of mHLA-DR and TNF-α release to monitor injury-induced immune alterations in critically ill patients. To our knowledge, none focused on the correlation between these two markers over the first week of ICU stay, and none addressed this question in large cohorts of critically ill non-septic patients [20,21]. Thus, the objective of the present biomarker-oriented study was to determine the correlation between both these tests and their association with nosocomial infections and death in a large cohort of septic, trauma and surgical patients.

## 2. Material and Methods

### 2.1. Design

This is an ancillary analysis of the REALISM study, a prospective longitudinal, single-center observational study conducted in the Anesthesiology and Intensive Care Department at the Edouard Herriot Hospital [22]. The objective of the REALISM study was to broadly assess immune parameters in a cohort of critically ill patients during the first two months after an injury and to correlate these findings with clinical data and outcomes in order to define immunosuppression in ICU patients better. The protocol for this study was published previously [23].

### 2.2. Patients

The inclusion criteria were: patients aged >18 years, clinical diagnosis of sepsis as defined by 2016 SEPSIS-3 consensus guidelines [1], severe trauma with injury severity score (ISS) >15 or surgical patients undergoing major surgery such as oesophagogastrectomy, bladder resection with Brickers’ reconstruction, cephalic pancreaticoduodenectomy and abdominal aortic aneurysm surgery by laparotomy. Exclusion criteria were any of the following: the presence of a preexistent condition or treatment that could influence patients’ immune status, pregnancy, institutionalized patients or inability to obtain informed consent.

A cohort of 175 healthy volunteers aged from 18 to 82 years (81 males and 94 females) was also recruited prospectively. In order to account for the possible influence of age and sex on immune parameters, the distribution of healthy volunteers was based on the age and sex demographic data for the French population in 2016. Written informed consent was obtained from every healthy volunteer and patient upon inclusion. If a patient was unable to consent directly, informed consent was obtained from the patient’s legally authorized representative and reconfirmed from the patient at the earliest opportunity. Patients’ demographics, comorbidities, diagnosis, severity and clinical outcome were prospectively collected. Longitudinal follow-up was performed for a period of 90 days. 

### 2.3. TNF Release and mHLA-DR Measurements

Clinical samples and data were collected three times during the first week after admission: on day 1 or 2 (D1–2), day 3 or 4 (D3–4) and day 5, 6 or 7 (D5–7). One sample was collected in healthy volunteers during the study visit, and clinical data were recorded. 

Ex vivo stimulation of whole blood by LPS was performed through the use of TruCulture tubes (MYRIAD RBM, Austin, TX, USA) containing a standardized LPS component. The tubes contain the medium alone (Null; Null-R; MYRIAD RBM) or the medium with LPS 100 ng/mL (LPS from Escherichia coli O55:B5) (LPS-R; MYRIAD RBM). The blood samples were collected on heparin and immediately transported to the laboratory, where 1 mL of heparinized blood was transferred to each TruCulture tube and incubated for 24 h at 37 °C. Following incubation, the supernatant (medium and plasma) was collected using a separation valve (according to manufacturer instructions) and stored at −80 °C until batch quantification of TNF-α by ELISA (BE55001; BL International-Tecan, Männedorf, Switzerland). 

The number of HLA-DR molecules per monocyte was determined using the BD Quantibrite Anti–HLA-DR/Anti-Monocyte standardized method (Becton Dickenson, Franklin Lakes, NJ, USA) as previously described [24].

### 2.4. Definition of Endpoint

The primary endpoint for this study was the adverse outcome, defined as the occurrence of death or a nosocomial infection within the 30 days following ICU admission and during the hospital stay. During the ICU stay, patients were screened daily for exposure to invasive devices (intubation, indwelling urinary catheter and central venous line) and occurrence of secondary infection. Information related to infections were collected, reviewed and validated by a blinded dedicated adjudication committee, comprising three physicians not involved in the recruitment of the patients, with confirmation of secondary infection made according to the definitions used by the European Centre for Disease Prevention and Control [25] and the Infectious Diseases Society of America.

### 2.5. Statistical Analysis

Qualitative variables are presented as numbers and percentages, and quantitative variables as median and 25th/75th percentiles. Chi-square or Fisher’s exact test was used for qualitative variables assessment. Quantitative variables were compared with the Mann–Whitney U test or Student’s *t*-tests according to the distribution of the variables. Normality was assessed using histograms and Shapiro–Wilk test. Receiver operating characteristic curves for mHLA-DR and TNF-α release measured at time point D5–D7 were estimated. Correlation between mHLA-DR and TNF-α release was assessed using Spearman’s rho correlation coefficients after log transformation of the data. The coefficients were calculated at the three time points and in all predefined subgroups. Kaplan–Meier estimations were performed at different time points for both immune parameters combined and individually. A log-rank test was applied. The Cox proportional hazards model was used at time points D5–D7 as the association was the strongest at this time in univariate analysis. Clinically relevant variables were included in the model, namely age, severity assessed by Sequential Organ Failure Assessment (SOFA) Score and invasive devices exposure duration (tracheal intubation, venous catheter, urinary catheter). Hazard ratios calculated for both immune parameters were normalized to an increment from the first to the third quartile to allow comparison between the two models. All statistical analyses were performed with R software v4.0.3.

## 3. Results

Three hundred and fifty-three patients were included: 107 septic patients, 137 trauma patients and 109 patients included after elective major surgery. The median age was 60 years (range 47–71). At inclusion, the median SOFA score was 5 (range 1–8). Of 353 patients, 74 (21%) developed at least one secondary infection within the first 30 days after enrolment. This incidence was 18.7%, 16.8% and 28.4% in sepsis, trauma and surgery patients, respectively. The mortality rate at day 30 in the whole cohort was 5%. This rate was higher among septic patients (16%). Additional results were provided elsewhere [22]. Regarding the occurrence of adverse outcomes, clinical and biological characteristics at inclusion and exposure to invasive devices are reported in Table 1. Characteristics related to septic, trauma and surgery subgroups are reported in Supplementary Tables S1–S3. Age-matched healthy volunteers (*n* = 175) were also recruited. 

**Figure 1:**
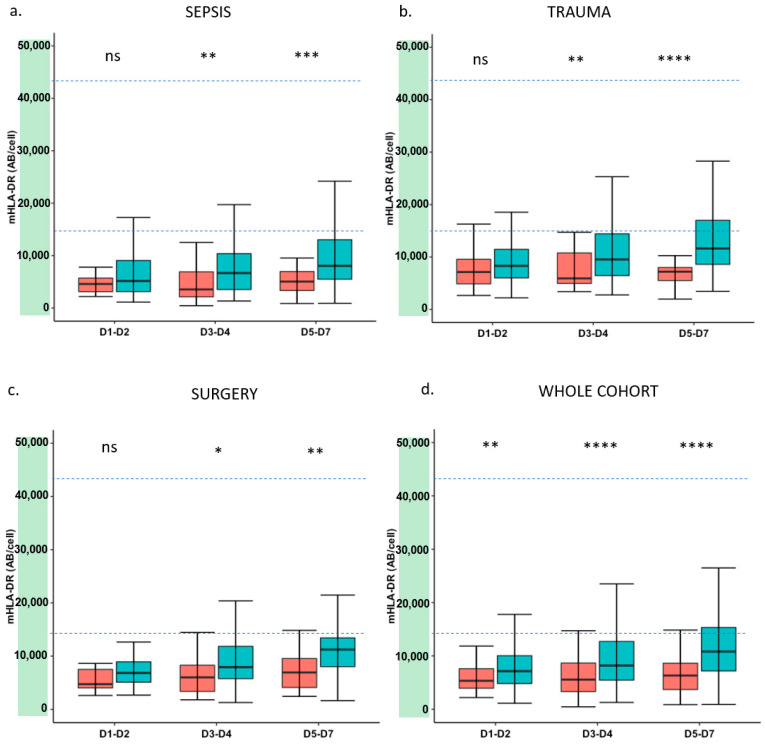
Time course of mHLA-DR expression after injury. (**a**) mHLA-DR expression in septic patients; (**b**) mHLA-DR expression in trauma patients; (**c**) mHLA-DR expression in surgical patients; (**d**) mHLA-DR expression in the whole cohort of patients. Results are presented as box plots and are expressed as numbers of anti-mHLA-DR antibodies bound per monocyte (AB/C). Dotted lines represent reference values for mHLA-DR, based on 2.5th and 97.5th percentiles of healthy volunteers’ values. Blue plots correspond to patients who did not present with clinical worsening (*n* = 263). Red plots correspond to patients with clinical worsening (*n* = 90). Mann–Whitney test used for comparison between groups. Ns—non-significant, * *p* < 0.05, ** *p* < 0.01, *** *p*< 0.001, **** *p* < 0.0001.

**Figure 2:**
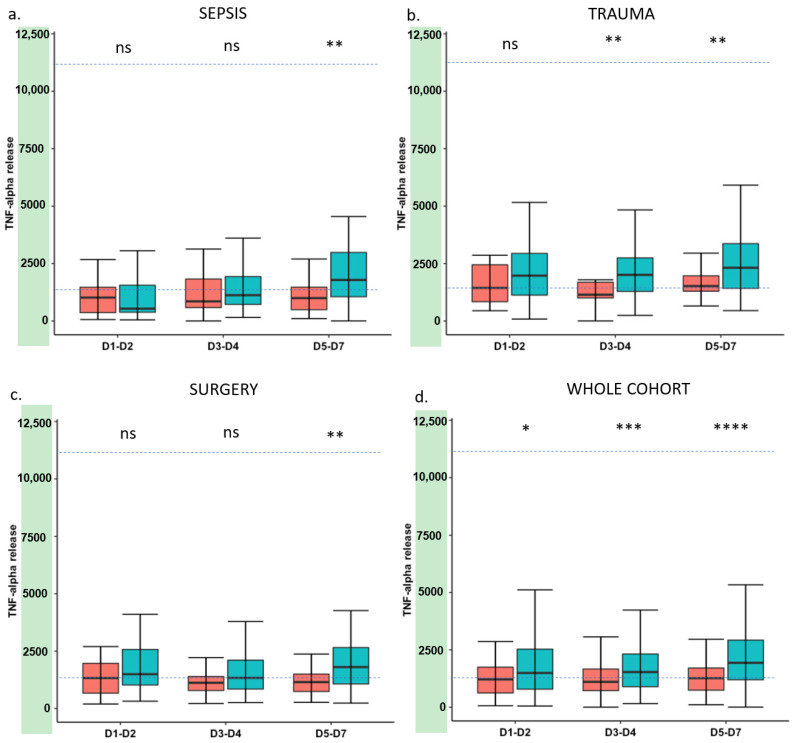
Time course of TNF-α release after injury. (**a**) TNF-α release in septic patients; (**b**) TNF-α release in trauma patients; (**c**) TNF-α release in surgical patients; (**d**) TNF-α release in the whole cohort of patients. Results are presented as box plots and are expressed as concentrations (pg/mL) of TNF-α in supernatants after whole blood LPS stimulation. Dotted lines represent reference values for TNF-α release, based on 2.5th and 97.5th percentiles of healthy volunteers’ values. Blue plots correspond to patients who did not present with clinical worsening (*n* = 263). Red plots correspond to patients with clinical worsening (*n* = 90). Mann–Whitney test was used for comparison between groups. Ns—non-significant, * *p* < 0.05, ** *p* < 0.01, *** *p*< 0.001, **** *p* < 0.0001.

Figure 1 shows mHLA-DR at each time point in the septic, trauma and surgery subgroups and in the whole cohort. All types of injuries induced a decrease in mHLA-DR expression compared to the healthy volunteers’ group. Overall, this decrease was more marked in the group of patients presenting with adverse outcomes. The difference between the two groups increased over time, reaching statistical significance at D3–D5 in all types of injury. The same tendency was observed for TNF-α release (Figure 2) even though the difference between the groups was less marked. 

**Figure 3:**
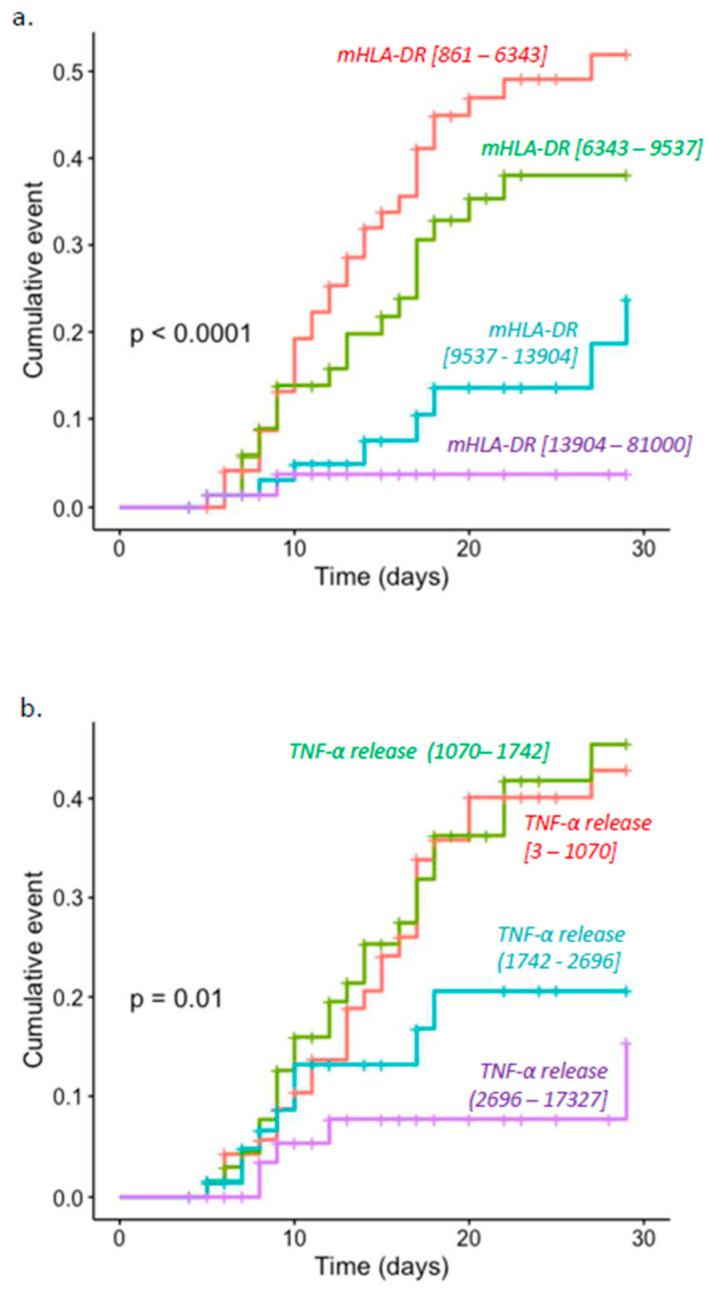
Cumulative incidence curves for clinical worsening at D30. Population was stratified into four groups depending on quartiles of each marker in the population at D5–D7. Cumulative incidence curves were estimated with Kaplan–Meier method, and log-rank test was applied. (**a**) mHLA-DR expression at D5–D7 (**b**) TNF-α release at D5–D7. Of note, cumulative incidence curves for clinical worsening at D30 based on mHLA-DR expression at D3–D4 are depicted in Supplementary Figure S1.

As previously described by principal component analyses [22], marker trajectories were similar between etiologies (i.e., the common response to injuries). As the association with adverse outcomes was more pronounced at D5–D7, we focused on this time point to next compare mHLA-DR and TNF release in the whole cohort. Kaplan–Meier analysis was used to provide unadjusted cumulative events for adverse outcomes at D30 with patients stratified into four groups based on quartiles of the range of values of each marker at D5–D7. The lowest expressions of both markers were associated with adverse outcomes (mHLA-DR, *p* < 0.001; TNF-α release, *p* = 0.01). Cumulative incidence curves are shown in Figure 3. Based on the ROC curves, mHLA-DR expression presented an area under the curve of 0.78 compared to 0.71 for TNF-α release. 

**Figure 4:**
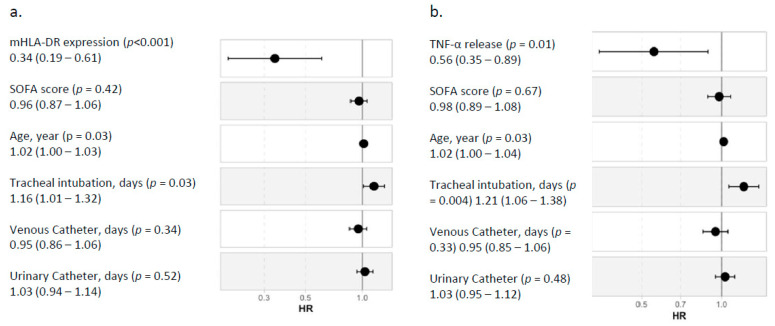
Multivariable analysis. Cox proportional hazards model at D5–D7 including (**a**) mHLA-DR expression or (**b**) TNF-α release. Hazard ratios calculated for both immune parameters were normalized to an increment from first to third quartile to allow comparison between the two models.

In multivariate analysis, after accounting for clinically relevant variables in the Cox model (age, SOFA, tracheal intubation, catheterization), elevated mHLA-DR and TNF-α release measured at D5–7 were significantly and independently associated with better outcomes with respective normalized hazard ratios of 0.34 and 0.56 (Figure 4). Age and the duration of invasive mechanical ventilation were also found as risk factors, whereas the SOFA score and exposure to other invasive devices (central or urinary catheter) were not. 

**Figure 5:**
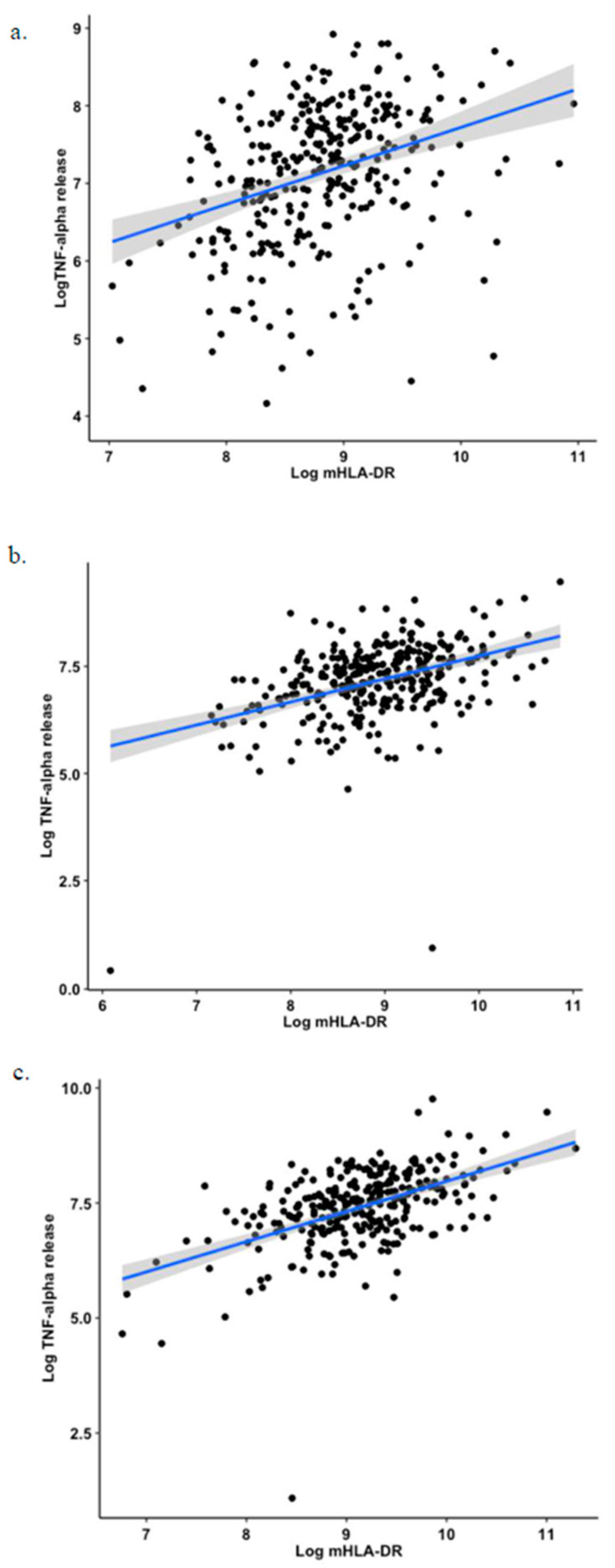
Correlation plots between mHLA-DR expression and TNF-α release. Correlation between log transformation of mHLA-DR expression and TNF-α release at (**a**) time points D1–D2, (**b**) time points D3–D4, (**c**) time points D5–D7 in whole cohort.

As both markers were separately associated with adverse outcomes, we then estimated the correlation between mHLA-DR and TNF release. Overall, although significant, correlation in the whole cohort of patients was moderate (i.e., ρ = 0.41, *p* < 0.001). In subgroup analysis, this correlation was the greatest in the sepsis group (ρ = 0.45, *p* < 0.001) and improved over time, i.e., the highest correlations were observed at D5–D7. All results are presented in Table 2 and Figure 5.

**Figure 6:**
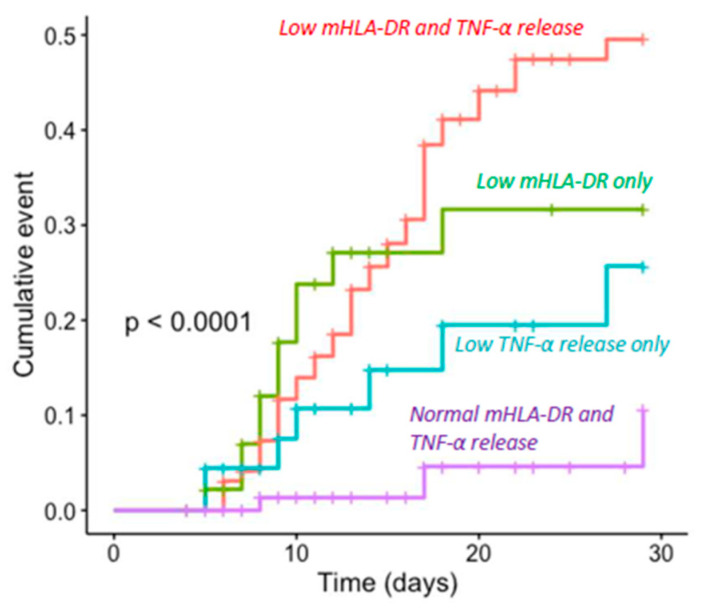
Cumulative incidence curve for clinical worsening at D30 after combining mHLA-DR expression and TNF-α release. Population was stratified in four groups at D5–D7 depending on alteration of one or both markers. Cumulative incidence curve was estimated with Kaplan–Meier methodology, and log-rank test was applied. mHLA-DR: human leukocyte antigen DR on circulating monocytes, TNF-α: Tumor necrosis factor α. Stratification was based on median distribution of each marker at D5–D7 (mHLA-DR = 9537 Ab/c and TNF-α release = 1741 pg/mL).

Finally, as correlation was moderate between both markers, we combined them to stratify patients into four groups at D5–D7. Cut-off thresholds were defined as TNF-α or mHLA-DR results below the median in the population at D5–D7 for each marker. Group 1 had both TNF-α release and mHLA-DR expression altered, Group 2 only had altered mHLA-DR expression, Group 3 only had altered TNF-α release and Group 4 had increased values of TNF-α release and mHLA-DR expression. The incidence of adverse outcomes in each group is reported in Table 3. When combining low values for both markers, we identified a subset of patients with a higher risk of worsening (41% of patients with an adverse outcome). Conversely, when only one marker was altered, the incidence of adverse outcome was much lower, ranging from 16% (low TNF only) and 24% (low mHLA-DR only) to 3% (both markers above median). Cumulative incidence curves related to each marker combination are reported in Figure 6. 

## 4. Discussion

In this ancillary analysis of the REALISM dataset [22], we showed that both decreased mHLA-DR and TNF-α release were associated with a higher risk of death or nosocomial infections in critically ill patients with sepsis, trauma or after elective surgery.

mHLA-DR and TNF-α release was markedly decreased immediately after ICU admission. After this initial response to stress and due to homeostatic mechanisms, some patients recover from their initial insult and present with a progressive rise in mHLA-DR and TNF-α release over time. In contrast, we observed that both markers remained significantly lower in the subgroup of patients with adverse outcomes. In addition, for both markers, the strength of the association was maximal at D5–7 compared to earlier time points. This is in accordance with previous studies, which did not observe any association upon ICU admission [25,26]. The existence of two overlapping pro-inflammatory and anti-inflammatory responses induced at the initial stage after injury may have blurred the signal of immunosuppression [2].

We observed that mHLA-DR and TNF-α release was statistically but moderately correlated. As they are focused on monocytes, both markers are interrelated, but they represent slightly different immune mechanisms. HLA-DR is part of the MHC class II molecule system, which allows monocytes to exert the function of antigen-presenting cells, thus linking innate immunity to adaptive immune response. Decreased mHLA-DR is thus associated with a reduced capacity of monocytes to induce T-cell responses [27]. Elsewhere, TNF-α release reflects monocyte inflammatory function. Decreased TNF-α release by monocytes was linked with the phenomenon of endotoxin tolerance [16]. However, as it is currently performed in whole blood, it cannot be excluded that part of the TNF-α release after LPS stimulation may be coming from other leukocytes, although this is likely to be a limited amount.

The present results confirm that there is an association between markers of immunosuppression and adverse outcome after various types of injury such as trauma or major surgery [8,11,28,29]. Our study also confirms the strong and independent association between each marker and adverse outcome after multivariate analysis. However, this association was stronger for mHLA-DR (Figure 1, Figure 2, Figure 3 and Figure 4 and Figure 6). Interestingly, when combining the two markers, we were able to enrich a subgroup of patients with a higher risk of adverse outcomes in which almost 50% of patients would deteriorate (Figure 6). As both markers may delineate different pathways of immune modifications, alterations of both of them may reflect a more pronounced state of immunosuppression, and thus, combining them could be helpful to identify patients that may benefit the most from the use of immunostimulatory drugs.

Unlike our work, two recent studies failed to establish a link between TNF-α release and complicated ICU course. In a cohort of septic shock patients, Drewry et al. [20] observed a trend toward lower values of TNF-α measured on days 6 to 8 after admission between survivors and non-survivors; however, the difference was not statistically different. To note, the number of patients analyzed at this time point was low (*n* = 28) and may account for this lack of statistical power. Likewise, Levin et al. [30] did not report any difference in rates of nosocomial infections and worse clinical outcomes among groups of patients based on stratification of TNF-α levels. However, they limited their analyses to the values measured on ICU admission, whereas our findings suggest that TNF-α values have better performance when measured at D5–D7. The lack of consistency in the association with clinical outcomes in some studies may also result from the lack of reproducibility in the measurement of TNF-α release, which has never been standardized [11]. This issue was underscored by Segre et al. [19]. They reported high variability in the values of TNF-α release depending on the LPS source, LPS concentration, duration and temperature of incubation, as well as sample handling before stimulation. As immune monitoring may be used to guide immunoadjuvant therapies in the future, this question needs to be resolved in order to identify which patients would benefit from such a strategy precisely. In the present study, we used standardized commercial tubes containing LPS from E. Coli O55:B5 in order to minimize the bias and variability introduced by sample shipping and manipulation. Conversely, mHLA-DR standardization was extensively reported [14] and has proven to ensure inter-laboratory reproducibility [24,31]. Pre-analytical handling requires EDTA anticoagulation, storage on ice as soon as possible and analysis by flow cytometry within 4 h after sampling. These conditions may limit the accessibility of the technique in centers that do not have rapid access to flow cytometry facilities [32]. Recent data nevertheless suggest the possibility to measure mHLA-DR after blood collection in stabilizing sampling tubes allowing to quantify mHLA-DR expression up to 72 h after sampling and storage at room temperature [31,33]. Alternatively, a bedside protocol for flow cytometry measurement of mHLA-DR was recently proposed [34]. This recent progress could help to overcome drawbacks in mHLA-DR measurement and facilitate its use.

Our study has several limitations that should be addressed. First, this was a mono-center study. That said, the patients’ recruitment was performed among three different ICUs and allowed us to investigate different types of injuries. Second, regarding ICU patients, the overall mortality rate of the cohort may be considered relatively low due to the inclusion of trauma and patients after elective surgery. This supports our choice to evaluate adverse outcomes as defined by the occurrence of death or a secondary infection as the primary outcomes. Thus, in the next larger cohorts, the validity of the present results regarding association with mortality should be further confirmed.

## 5. Conclusions

We reported that both persistent decreased mHLA-DR and TNF-α release, two markers of immunosuppression, were significantly associated with adverse outcomes in a large cohort of patients recruited after different types of injuries. Overall, although mHLA-DR appears to be a more robust and standardized parameter, each marker can be used individually as a surrogate of immunosuppression in ICU patients, depending on each center’s facility. In addition, although a significant correlation was observed between markers, the present work illustrates that combining these two parameters could be of interest to identify a subgroup of patients with deep injury-induced immunosuppression better. This last aspect deserves further exploration. The development of robust, standardized and routinely available markers of injury-induced immunosuppression will help to promote wider use of reliable immune monitoring in the future. This is of major importance to guide future clinical trials evaluating immune-adjuvant treatments in critically ill patients.

## Figures and Tables

**Table 1:** Patients’ characteristics at inclusion.

	Adverse Outcome (*n* = 90)	Favorable Outcome (*n* = 263)	*p*-Value
Gender (*n*, %)	57 (63.3)	174 (66.2)	0.720
Age (years)	67.5 (55.3–75.0)	57.0 (44.0–70.0)	<0.001
BMI (kg/m^2^)	26.1 (22.2–29.9)	24.7 (22.3–27.6)	0.093
**Severity scores**			
SAPSII	35.0 (26.3–49.8)	26.0 (18.0–40.0)	<0.001
Charlson	2.0 (0.0–3.0)	1.0 (0.0–2.0)	<0.001
SOFA	7.0 (2.0–10.0)	4.0 (1.0–8.0)	<0.001
**Biological values**			
ALT (UI/L)	84.0 (41.8–150.0)	63.0 (32.0–160.0)	0.419
AST (UI/L)	126.0 (50.5–235.0)	83.0 (48.0–177.0)	0.189
Bilirubin (µmol/L)	17.5 (11.8–34.0)	15.0 (9.0–35.0)	0.335
Creatinine (µmol/L)	108.5 (71.8–183.8)	87.0 (66.3–125.8)	0.016
Leucocytes (×10^9^/L)	13.9 (10.1–18.4)	12.7 (10.32–16.4)	0.389
Lymphocytes (×10^9^/L)	1.1 (0.8–1.6)	1.3 (0.82–1.8)	0.059
Monocytes (×10^9^/L)	0.9 (0.6–1.4)	0.9 ( 0.67–1.3)	0.709
Neutrophils (×10^9^/L)	11.6 (8.3–15.9)	10.7 (7.88–14.1)	0.271
Platelets (×10^9^/L)	193 (144–241)	206 (162.00–274)	0.112
PaO_2_/FiO_2_ (mm Hg)	242 (160–316)	246 (181–358)	0.146
Hemoglobin (g/dL)	113(93–127)	117 (103–132)	0.075
pH	7.34 (7.28, 7.39)	7.36 (7.30–7.41)	0.116
Lactate (mmol/L)	2.50 (1.70, 3.50)	2.20 (1.60–3.20)	0.205
**Organ failure**			
Coma (*n*, %)	7 (7.8)	19 (7.2)	1.000
Vasopressors (*n*, %)	57 (63)	121 (46)	0.007
Renal Replacement Therapy (*n*, %)	30 (33)	31 (12)	<0.001
**Exposure to invasive devices**			
Urinary Catheter (*n*, %)	81 (90)	213 (81)	0.070
Venous Catheter (*n*, %)	74 (82)	145 (55)	<0.001
Tracheal intubation (*n*, %)	59 (66)	105 (40)	<0.001
Invasive Ventilation D30 Free Days	17.00 (0.00, 28.75)	29.00 (27.00, 29.00)	<0.001
Urinary Catheter D30 Free Days	7(0–22)	27 (22–28)	<0.001
Venous Catheter D30 Free Days	0 (0–15)	23.00 (17–27)	<0.001
**Follow-up**			
Length of ICU stay	9.50 (4.00, 15.00)	5.00 (3.00, 8.00)	<0.001
Time to adverse outcome (days)	9.00 (5.00, 14.00)	NA	

Results are expressed as medians and interquartile ranges [IQR] or numbers and percentages (%). ALT: alanine transaminase, AST: Aspartate transaminase, BMI: Body mass index, SAPS: Simplified Acute Physiology Score, SOFA: Sequential Organ Failure Assessment. Chi-square or Fisher’s exact test was used for qualitative variables assessment. Quantitative variables were compared with Mann–Whitney U test or Student’s *t*-tests according to the distribution of the variables. Normality was assessed using histograms and Shapiro–Wilk test.

**Table 2:** Spearman’s correlation for mHLA-DR expression and TNF-α release.

Population	ρ	*p*-Value
All cohort (all time points)	0.41	<0.001
D1–D2	0.36	<0.001
D3–D4	0.39	<0.001
D5–D7	0.49	<0.001
Sepsis (all time points)	0.45	<0.001
Sepsis D1–D2	0.32	0.003
Sepsis D3–D4	0.40	<0.001
Sepsis D5–D7	0.52	<0.001
Trauma (all time points)	0.34	<0.001
Trauma D1–D2	0.17	0.04
Trauma D3–D4	0.36	<0.001
Trauma D5–D7	0.46	<0.001
Surgery (all time points)	0.38	<0.001
Surgery Day 1–2	0.39	<0.001
Surgery D3–D4	0.24	0.02
Surgery D5–D7	0.43	<0.001

**Table 3:** Incidence of clinical worsening after stratifying patients by combining mHLA-DR expression and TNF-α release at D5–D7.

	Clinical Worsening	No Clinical Worsening
Group 1 (mHLA-DR expression and TNF-α release lower than medians)	40 (41%)	57 (59%)
Group 2 (only mHLA-DR expression lower than median)	11 (24%)	35 (76%)
Group 3 (only TNF-α release lower than median)	7 (16%)	38 (84%)
Group 4 (mHLA-DR expression and TNF- α release higher than medians)	3 (3%)	94 (97%)

Results are expressed as numbers and percentages (%). mHLA-DR: human leukocyte antigen DR on circulating monocytes, TNF-α: Tumor necrosis factor α. Stratification was based on median distribution of each marker at D5–D7 (mHLA-DR = 9537 Ab/c and TNF-α release = 1741 pg/mL). *p* < 0.0001.

## Data Availability

The original contributions presented in the study are included in the article/Supplementary Material, further inquiries can be directed to corresponding author.

## Supplementary Materials

The following are available online at https://www.mdpi.com/article/10.3390/jcm11010096/s1, Figure S1: Cumulative incidence curves for clinical worsening at D30 stratified on mHLA-DR at D3-D4, Table S1: Patients’ characteristics at inclusion (sepsis cohort), Table S2: Patients’ characteristics at inclusion (trauma cohort), Table S3: Patients’ characteristics at inclusion (surgery cohort).

## Realism Study Group

HCL: Sophie ARNAL, Caroline AUGRIS-MATHIEU, Frédérique BAYLE, Liana CARUSO, Charles-Eric BER, Asma BEN-AMOR, Anne-Sophie BELLOCQ, Farida BENATIR, Anne BERTIN-MAGHIT, Marc BERTIN-MAGHIT, André BOIBIEUX, Yves BOUFFARD, Jean-Christophe CEJKA, Valérie CERRO, Jullien CROZON-CLAUZEL, Julien DAVIDSON, Sophie DEBORD-PEGUET, Benjamin DELWARDE, Robert DELEAT-BESSON, Claire DELSUC, Bertrand DEVIGNE, Laure FAYOLLE-PIVOT, Alexandre FAURE, Bernard FLOCCARD, Julie GATEL, Charline GENIN, Thibaut GIRARDOT, Arnaud GREGOIRE, Baptiste HENGY, Laetitia HURIAUX, Catherine JADAUD, Alain LEPAPE, Véronique LERAY, Anne-Claire LUKASZEWICZ, Guillaume MARCOTTE, Olivier MARTIN, Marie MATRAY, Delphine MAUCORT-BOULCH, Pascal MEURET, Céline MONARD, Florent MORICEAU, Guillaume MONNERET, Nathalie PANEL, Najia RAHALI, Thomas RIMMELE, Cyrille TRUC, Thomas UBERTI, Hélène VALLIN, Fabienne VENET, Sylvie TISSOT, Abbès ZADAM. BioMérieux: Sophie BLEIN, Karen BRENGEL-PESCE, Elisabeth CERRATO, Valérie CHEYNET, Emmanuelle GALLET-GORIUS, Audrey GUICHARD, Camille JOURDAN, Natacha KOENIG, François MALLET, Boris MEUNIER, Virginie MOUCADEL, Marine MOMMERT, Guy ORIOL, Alexandre PACHOT, Estelle PERONNET, Claire SCHREVEL, Olivier TABONE, Julien TEXTORIS, Javier YUGUEROS MARCOS. BIOASTER: Jérémie BECKER, Frédéric BEQUET, Yacine BOUNAB, Florian BRAJON, Bertrand CANARD, Muriel COLLUS, Nathalie GARCON, Irène GORSE, Cyril GUYARD, Fabien LAVOCAT, Philippe LEISSNER, Karen LOUIS, Maxime MISTRETTA, Jeanne MORINIERE, Yoann MOUSCAZ, Laura NOAILLES, Magali PERRET, Frédéric REYNIER, Cindy RIFFAUD, Mary-Luz ROL, Nicolas SAPAY, Trang TRAN, Christophe VEDRINE. SANOFI: Christophe CARRE, Pierre CORTEZ, Aymeric DE MONFORT, Karine FLORIN, Laurent FRAISSE, Isabelle FUGIER, Sandrine PAYRARD, Annick PELERAUX, Laurence QUEMENEUR. ESPCI: Andrew GRIFFITHS, Stephanie TOETSCH. GSK: Teri ASHTON, Peter J. GOUGH, Scott B. BERGER, David GARDINER, Iain GILLESPIE, Aidan MACNAMARA, Aparna RAYCHAUDHURI, Rob SMYLIE, Lionel TAN, Craig TIPPLE.

## Abbreviations

ICU	intensive care unit
LPS	lipopolysaccharide
mHLA-DR	human leukocyte antigen DR on circulating monocytes
SOFA	Sequential Organ Failure Assessment
TNF-α	Tumor necrosis factor α
WB	Whole blood
