# Supplementary material for: Concomitant Assessment of Monocyte HLA-DR Expression and Ex Vivo TNF-α Release as Markers of Adverse Outcome after Various Injuries—Insights from the REALISM Study
Source: J Clin Med. 2021 Dec 24;11(1):96. doi: 10.3390/jcm11010096 (PMC8745266; doi:10.3390/jcm11010096)
Supplement: Supplementary file 1 [file jcm-11-00096-s001.zip › jcm-1510955-supplementary.pdf]

Online supplementary material

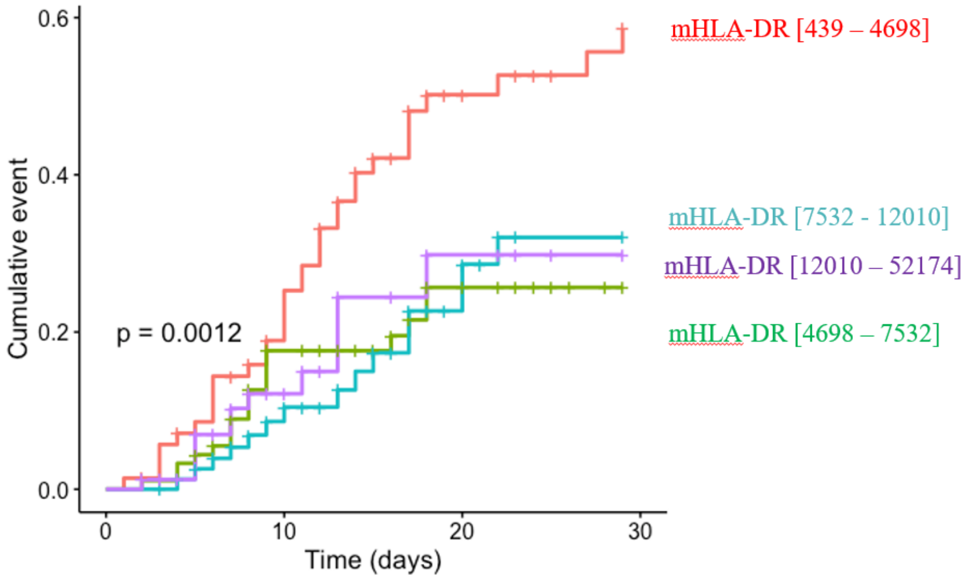

Figure S1. Cumulative incidence curves for clinical worsening at D30 stratified on mHLA-DR at D3-D4. Population was stratified into four groups depending on quartiles of each marker in the population at the given timepoint. Cumulative incidence curves were estimated with Kaplan-Meier method and log-rank test was applied.

Table S1. Patients' characteristics at inclusion (sepsis cohort).

	Clinical worsening (n = 35)	No clinical worsening (n = 72)	p-value
Gender, male (n, %)	22 (62.9)	47 (65.3)	0.976
Age	71.00 [63.00, 78.00]	67.00 [56.75, 77.00]	0.222
BMI	26.45 [22.98, 30.36]	25.43 [22.52, 30.31]	0.488
**Severity scores**			
SAPSII	50.00 [44.00, 57.50]	46.00 [35.75, 52.00]	0.033
Charlson	2.00 [2.00, 3.00]	2.00 [0.00, 4.00]	0.246
SOFA	10.00 [8.00, 11.50]	8.50 [7.00, 10.00]	0.054
**Biological values**			
ALT	57.00 [21.00, 116.50]	37.50 [21.50, 66.50]	0.250
AST	137.00 [48.50, 280.00]	60.00 [39.00, 149.50]	0.126
Bilirubin	19.00 [12.50, 45.25]	19.00 [11.50, 40.00]	0.932
Creatinin	193.00 [100.00, 262.50]	142.00 [99.00, 209.50]	0.176
Leucocytes	13.70 [8.83, 21.36]	12.63 [8.35, 22.34]	0.752
Lymphocytes	0.73 [0.38, 1.57]	1.04 [0.67, 1.46]	0.131
Monocytes	0.88 [0.54, 1.24]	0.70 [0.47, 1.43]	0.848
Neutrophils	12.19 [7.98, 20.23]	11.57 [7.28, 20.42]	0.825
Platelets	188.00 [136.25, 264.25]	219.00 [150.50, 308.00]	0.534
PaO2/FiO2	185.50 [117.00, 278.25]	218.00 [174.00, 294.00]	0.161
Hemoglobin	109.00 [91.00, 124.50]	112.00 [93.00, 128.00]	0.662
pH	7.35 [7.28, 7.40]	7.37 [7.32, 7.43]	0.075
Lactate	2.70 [1.70, 3.90]	2.20 [1.70, 3.32]	0.142
**Organ failures**			
Coma (n, %)	2 (5.7)	4 (5.6)	1.000
Vasopressors (n, %)	3 (8.6)	5 (6.9)	1.000
Renal Replacement Therapy (n, %)	22 (62.9)	20 (27.8)	0.001
**Exposition to invasive devices**			
Urinary Catheter (n, %)	35 (100.0)	61 (84.7)	0.036
Venous Catheter (n, %)	34 (97.1)	63 (87.5)	0.210
Tracheal intubation (n, %)	29 (82.9)	47 (65.3)	0.098
Invasive Ventilation D30 Free Days	0.00 [0.00, 25.00]	27.00 [26.00, 29.00]	<0.001
Urinary Catheter D30 Free Days	0.00 [0.00, 7.00]	23.00 [15.00, 27.00]	<0.001
Venous Catheter D30 Free Days	0.00 [0.00, 0.00]	19.00 [11.50, 24.00]	<0.001

Results are expressed as median (IQR) or n (%). ALT: alanine transaminase, AST: Aspartate transaminase, BMI: Body mass index, SAPS: Simplified Acute Physiology Score, SOFA: Sequential Organ Failure Assessment.

Table S2. Patients' characteristics at inclusion (trauma cohort).

	**Clinical worsening (*n* = 24)**	**No clinical worsening (*n* = 113)**	***p*-value**
Gender, male (*n*, %)	15 (62.5)	88 (77.9)	0.186
Age	52.50 [38.00, 64.75]	46.00 [28.00, 56.00]	0.080
BMI	27.00 [23.11, 30.50]	24.89 [22.56, 27.57]	0.162
**Severity scores**			
SAPSII	34.50 [28.50, 45.25]	24.00 [19.00, 34.00]	0.001
Charlson	0.00 [0.00, 0.25]	0.00 [0.00, 0.00]	0.346
SOFA	7.00 [1.00, 9.25]	2.00 [0.00, 7.00]	0.014
**Biological values**			
ALT	78.50 [50.75, 142.00]	68.00 [33.50, 159.00]	0.592
AST	124.00 [86.00, 186.75]	83.50 [49.00, 199.50]	0.405
Bilirubin	13.00 [12.50, 22.00]	10.00 [7.00, 14.00]	0.244
Creatinin	89.00 [66.25, 119.50]	81.00 [68.00, 97.50]	0.317
Leucocytes	14.41 [10.55, 17.16]	13.62 [10.66, 16.99]	0.837
Lymphocytes	1.26 [0.97, 1.76]	1.32 [0.91, 2.06]	0.635
Monocytes	1.02 [0.75, 1.50]	1.00 [0.77, 1.29]	0.579
Neutrophils	11.62 [8.21, 14.85]	11.06 [8.06, 14.09]	0.739
Platelets	190.50 [139.50, 220.25]	196.00 [158.00, 248.00]	0.375
PaO2/FiO2	296.00 [203.25, 352.25]	286.00 [196.75, 405.00]	0.816
Hemoglobin	116.50 [100.25, 137.75]	123.00 [111.50, 138.00]	0.264
pH	7.34 [7.30, 7.38]	7.36 [7.30, 7.39]	0.651
Lactate	2.10 [1.60, 2.90]	2.40 [1.50, 3.15]	0.972
**Organ failures**			
Coma (*n*, %)	5 (20.8)	9 (8.0)	0.129
Vasopressors (*n*, %)	15 (62.5)	42 (37.2)	0.040
Renal Replacement Therapy (*n*, %)	6 (25.0)	5 (4.4)	0.003
**Exposition to invasive devices**			
Urinary Catheter (*n*, %)	23 (95.8)	80 (70.8)	0.020
Venous Catheter (*n*, %)	7 (29.2)	73 (64.6)	0.003
Tracheal intubation (*n*, %)	19 (79.2)	51 (45.1)	0.005
Invasive Ventilation D30 Free Days	19.00 [3.00, 29.00]	29.00 [28.00, 29.00]	<0.001
Urinary Catheter D30 Free Days	0.00 [0.00, 18.50]	26.00 [23.00, 28.00]	<0.001
Venous Catheter D30 Free Days	15.00 [0.00, 19.75]	27.00 [25.00, 29.00]	<0.001

Results are expressed as median (IQR) or n (%). ALT: alanine transaminase, AST: Aspartate transaminase, BMI: Body mass index, SAPS: Simplified Acute Physiology Score, SOFA: Sequential Organ Failure Assessment.

Table S3. Patients' characteristics at inclusion (surgery cohort).

	Clinical worsening (n = 31)	No clinical worsening (n = 78)	p-value
Gender, male (n, %)	20 (64.5)	39 (50.0)	0.246
Age	70.00 [62.00, 72.50]	63.00 [55.00, 72.75]	0.043
BMI	25.39 [21.59, 27.96]	24.20 [21.97, 26.60]	0.513
**Severity scores**			
SAPSII	26.00 [20.00, 31.00]	18.00 [14.00, 23.00]	0.001
Charlson	3.00 [2.00, 3.00]	2.00 [2.00, 3.00]	0.017
SOFA	2.00 [1.00, 5.00]	1.00 [0.25, 3.75]	0.090
**Biological values**			
ALT	166.00 [78.00, 207.00]	100.00 [63.75, 276.75]	0.682
AST	124.00 [62.00, 150.00]	115.50 [66.50, 176.25]	0.843
Bilirubin	18.00 [10.00, 27.00]	14.00 [10.00, 27.00]	0.830
Creatinin	82.00 [61.00, 127.00]	71.00 [53.00, 105.00]	0.199
Leucocytes	13.73 [10.58, 18.08]	12.42 [10.67, 15.01]	0.179
Lymphocytes	1.10 [0.87, 1.48]	1.26 [0.91, 1.82]	0.389
Monocytes	1.00 [0.69, 1.37]	0.97 [0.73, 1.19]	0.668
Neutrophils	10.55 [8.44, 14.82]	10.25 [8.39, 12.07]	0.184
Platelets	207.00 [155.00, 243.00]	224.00 [188.00, 269.75]	0.126
PaO2/FiO2	293.00 [253.00, 318.00]	341.00 [315.50, 398.50]	0.124
Hemoglobin	111.50 [93.00, 124.00]	108.00 [100.00, 125.75]	0.732
pH	7.33 [7.30, 7.36]	7.35 [7.31, 7.40]	0.446
Lactate	2.90 [2.45, 2.95]	2.00 [1.75, 2.75]	0.310
**Organ failures**			
Coma (n, %)	0	0	
Vasopressors (n, %)	10 (32.3)	12 (15.4)	0.086
Renal Replacement Therapy (n, %)	2 (6.5)	6 (7.7)	1.000
**Exposition to invasive devices**			
Urinary Catheter (n, %)	23 (74.2)	72 (92.3)	0.026
Venous Catheter (n, %)	23 (74.2)	42 (53.8)	0.082
Tracheal intubation (n, %)	11 (35.5)	7 (9.0)	0.002
Invasive Ventilation D30 Free Days	29.00 [28.50, 29.00]	29.00 [29.00, 29.00]	0.456
Urinary Catheter D30 Free Days	24.00 [20.50, 26.50]	28.00 [26.00, 29.00]	<0.001
Venous Catheter D30 Free Days	10.00 [4.00, 23.00]	23.00 [18.00, 26.00]	0.004

Results are expressed as median (IQR) or n (%). ALT: alanine transaminase, AST: Aspartate transaminase, BMI: Body mass index, SAPS: Simplified Acute Physiology Score, SOFA: Sequential Organ Failure Assessment.
